# How Does Ginsenoside Rh2 Mitigate Adipogenesis in Cultured Cells and Obese Mice?

**DOI:** 10.3390/molecules25102412

**Published:** 2020-05-21

**Authors:** Longyun Zhang, Carlos Virgous, Hongwei Si

**Affiliations:** 1 Department of Human Sciences, Tennessee State University, Nashville, TN 37209, USA; geniisofla@gmail.com; 2 Animal Care Facility, Meharry Medical College, Nashville, TN 37208, USA; cvirgous@mmc.edu

**Keywords:** ginsenoside Rh2, adipogenesis, PPAR-γ pathway, preadipocytes, obese mice

## Abstract

Ginsenoside Rh2, an intermediate metabolite of ginseng, but not naturally occurring, has recently drawn attention because of its anticancer effect. However, it is not clear if and how Rh2 inhibits preadipocytes differentiation. In the present study, we hypothesized that ginsenoside Rh2 attenuates adipogenesis through regulating the peroxisome proliferator-activated receptor gamma (PPAR-γ) pathway both in cells and obese mice. Different concentrations of Rh2 were applied both in 3T3-L1 cells and human primary preadipocytes to determine if Rh2 inhibits cell differentiation. Dietary Rh2 was administered to obese mice to determine if Rh2 prevents obesity in vivo. The mRNA and protein expression of PPAR-γ pathway molecules in cells and tissues were measured by real-time polymerase chain reaction (RT-PCR) and Western blot, respectively. Our results show that Rh2 dose-dependently (30–60 μM) inhibited cell differentiation in 3T3-L1 cells (44.5% ± 7.8% of control at 60 μM). This inhibitory effect is accompanied by the attenuation of the protein and/or mRNA expression of adipogenic markers including PPAR-γ and CCAAT/enhancer binding protein alpha, fatty acid synthase, fatty acid binding protein 4, and perilipin significantly (*p* < 0.05). Moreover, Rh2 significantly (*p* < 0.05) inhibited differentiation in human primary preadipocytes at much lower concentrations (5–15 μM). Furthermore, dietary intake of Rh2 (0.1 g Rh2/kg diet, w/w for eight weeks) significantly (*p* < 0.05) reduced protein PPAR-γ expression in liver and hepatic glutathione reductase and lowered fasting blood glucose. These results suggest that ginsenoside Rh2 dose-dependently inhibits adipogenesis through down-regulating the PPAR-γ pathway, and Rh2 may be a potential agent in preventing obesity in vivo.

## 1. Introduction

Ginseng (*Panax ginseng*) has been used as a medicinal herb and functional food in Asia for over 2000 years [1], and it is the fifth most commonly used natural product in the United States [2]. There are four major ginsengs according to the preparation method: fresh ginseng, white ginseng (dehydration of fresh ginseng using sunlight), red ginseng (steaming dry/fresh ginseng at 95–100 °C for a reasonable time), and black ginseng (nine-time repetitive steaming white ginseng at 95–100 °C for 3 h) [3]. Red ginseng, the most attractive ginseng, accounting 59% in the South Korean market, is believed to have better health benefits than fresh ginseng and white ginseng, because more saponins and ginsenosides—the bioactive components of ginseng—are produced by the heating process. Particularly, ginsenosides such as Rg3 and Rh2, which are trace or not detectable in fresh or white ginseng, are dramatically increased by this process in red ginseng. This ginsenoside Rg3 can be transformed to ginsenoside Rh2 by intestinal bacteria or thermal process [4,5,6]. Both Rg3 and Rh2 have been reported to have special health benefits, including anticancer [7,8,9], anti-diabetes [10,11], anti-Alzheimer disease [12], and anti-inflammation [13].

Recently, the anti-obesity effect of ginseng has been extensively investigated [14] because of the worldwide fast increasing obesity prevalence. For instance, Korean red ginseng extracts (200 mg/kg, i.p. for three weeks) reduced food intakes and decreased the levels of leptin and neuropeptide Y in high-fat diet-fed rats [15]. American ginseng berry juice (oral gavage, once a day, 0.6 mL/kg for 10 days) reduced body weight gain in mice [16]. While the anti-obesity effects of ginseng crude extracts/juice have been reported in cells and animals, the only two reports of Rh2 on obesity are controversial. Rh2 promotes fat accumulation at low concentrations (0.01–1 μM) by activating glucocorticoid receptor in 3T3-L1 cells [17]; however, at a high level (at 20 and 40 μM), Rh2 attenuates 3T3-L1 cell differentiation [18]. This cell differentiation, named adipogenesis, plays a vital role in adult and childhood obesity development [19,20]. In the present study, we hypothesized that ginsenoside Rh2 inhibits adipogenesis through attenuating the peroxisome proliferator-activated receptor gamma (PPAR-γ) pathway in preadipocytes. The objectives of the present study were to determine if (1) Rh2 dose-dependently attenuates preadipocytes differentiation both in rodent preadipocytes (3T3-L1 cells) and human primary preadipocytes (HPPs); (2) dietary Rh2 intake prevents adipogenesis in obese mice; and (3) Rh2 attenuates adipogenesis by PPAR-γ and CCAAT/enhancer binding protein (C/EBP) pathway both in vitro and in vivo.

## 2. Results

### 2.1. Ginsenoside Rh2 Dose-Dependently Suppresses 3T3-L1 Differentiation without Cytotoxicity

**Figure 1:**
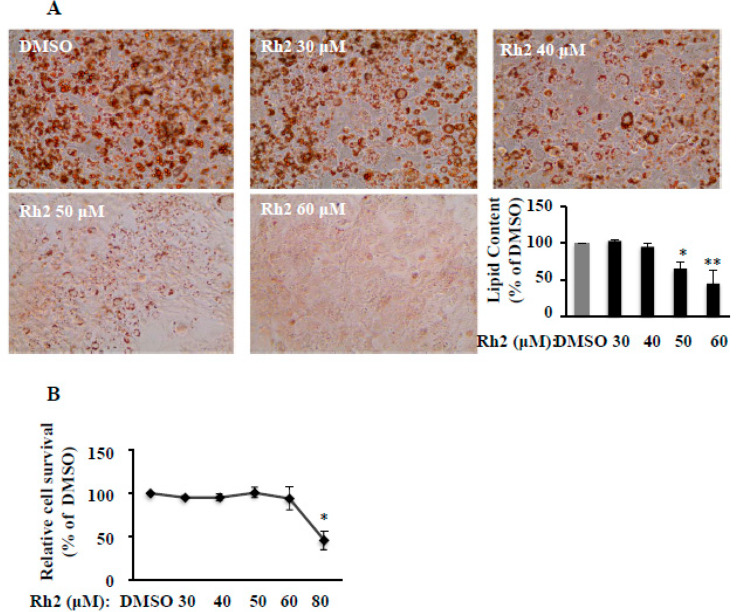
Ginsenoside Rh2 dose-dependently inhibits cell differentiation in 3T3-L1 cells. The cells were induced to differentiation by 3-isobutyl-1-methylxanthine (IBMX) + dexamethasone + insulin (MDI) mixture with/out Rh2 for seven days, and then stained with Oil-red O and dissolved in isopropanol. The relative fat accumulation was measured by a Synergy H1 hybrid reader at 490 nm. Oil-Red O representative images of lipid accumulation and the bar graphs are shown (**A**, 200 × magnification). A cell toxicity study was measured by an MTT assay (**B**). Values are means ± SE, *n* = 4. * *p* < 0.05, ** *p* < 0.01 vs. dimethyl sulfoxide (DMSO).

Ginsenoside Rh2 (30, 40, 50, or 60 μM) was added in the medium from day 0 (adding differential inducer 3-isobutyl-1-methylxanthine (IBMX) + dexamethasone + insulin (MDI)) and replaced on day 3 and day 5 in 3T3-L1 cells. On day 7, cells were stained with Oil-Red O to take images or extract fat by adding 100% isopropanol and measured the absorbance at 490 nm to evaluate the fat accumulation. As showed in Figure 1A, Rh2 inhibited MDI-induced intracellular fat droplets formation dose-dependently. At 60 µM, Rh2 reduced fat accumulation by up to 46% of the dimethyl sulfoxide (DMSO) control. We did not show the data on concentrations of 1 µM, 10 µM, and 20 µM because there were no significant effects on differentiation at these concentrations. The toxicity of ginsenoside Rh2 was evaluated by the cell toxicity MTT assay. We found that there was no significant effect on cell viability at the selected level of ginsenoside Rh2 (10–60 µM) in 3T3-L1 cells (Figure 1B). However, cells were significantly killed at 80 µM, which is in line with the previous report that ginsenoside Rh2 exerted cellular toxicity at doses of 80–160 µM [18]. These results indicate that ginsenoside Rh2 can prevent preadipocytes differentiation in a dose-dependent manner without cytotoxicity.

### 2.2. Ginsenoside Rh2 Dose-Dependently Inhibits PPAR-γ and C/EBP-α Protein Expressions in 3T3-L1 Cells

**Figure 2:**
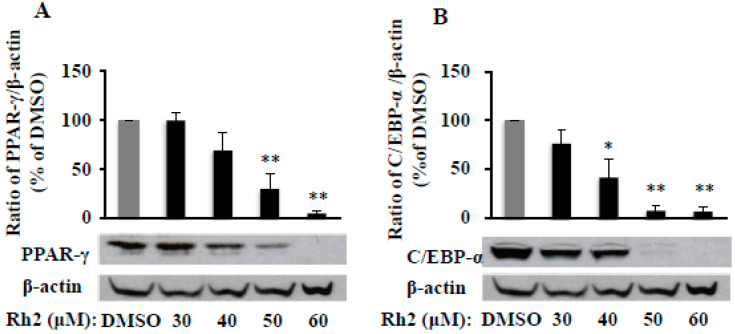
Ginsenoside Rh2 dose-dependently suppresses protein expressions of PPAR-γ (**A**) and CCAAT/enhancer binding protein (C/EBP)-α (**B**) in 3T3-L1 cells. On day 7, cells treated with various concentrations of Rh2 were collected to measure peroxisome proliferator-activated receptor gamma (PPAR-γ) and C/EBP-α protein expressions by Western blotting and normalized by β-actin expression. Values are means ± SE, *n* = 3. A set of representative images and bar graphs are shown. * *p* < 0.05, ** *p* < 0.01 vs. DMSO.

PPAR-γ and C/EBP-α are the two transcriptional factors of preadipocyte differentiation, and Rh2 suppressed 3T3-L1cells differentiation as above, we want to know if Rh2 affects protein level of PPAR-γ and C/EBP-α during the differentiation process. The Western blot results showed that MDI-induced PPAR-γ (Figure 2A) and C/EBP-α (Figure 2B) protein expressions were dose-dependently reduced by Rh2 in 3T3-L1 cells, the same pattern of the inhibitory effect of Rh2 in fat accumulation (Figure 1A). Particularly, protein expressions of PPAR-γ (Figure 2A) and C/EBP-α (Figure 2B) were significantly reduced to 4.9% (*p* < 0.01) and 6.5% (*p* < 0.01) of DMSO, respectively, by Rh2 at 60 μM. Therefore, ginsenoside Rh2 attenuates PPAR-γ and C/EBP-α protein expression, thereby inhibiting the adipogenesis process.

### 2.3. Ginsenoside Rh2 Abolishes MDI-Induced PPAR-γ mRNA Expression in 3T3-L1 Cells

**Figure 3:**
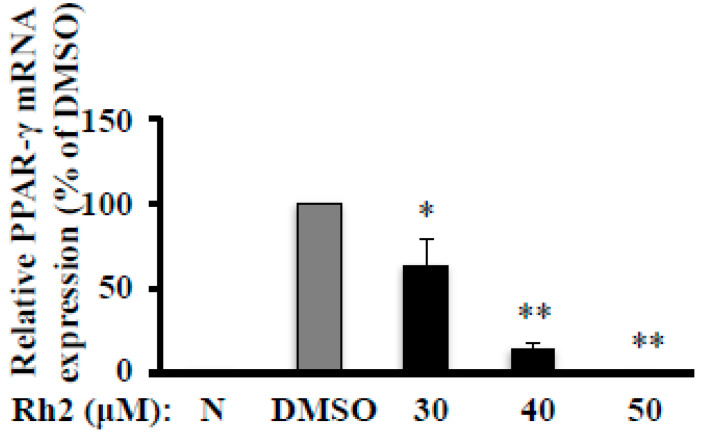
Ginsenoside Rh2 reduces PPAR-γ mRNA expression in 3T3-L1 cells. On day 7, cells treated with various concentrations of Rh2 were collected to measure PPAR-γ mRNA expression by quantitative real-time polymerase chain reaction (PCR) and normalized by β-actin expression. Values are means ± SE, *n* = 3. * *p* < 0.05, ** *p* < 0.01 vs. DMSO.

Although Rh2 abolished MDI-induced PPAR-γ protein expression, it is worth investigating whether the inhibitory effect of Rh2 on this key molecule is via a transcriptional mechanism. We measured PPAR-γ mRNA expression in 3T3-L1 cells using quantitative real-time polymerase chain reaction (PCR). Our results showed that Rh2 dose-dependently inhibited MDI-increased PPAR-γ mRNA expression after exposing of 3T3-L1 cells to various concentrations of Rh2 for seven days, notably reduced to 9.6% of DMSO at 50 μM (Figure 3). This effect is very consistent with its impact on fat accumulation (Figure 1A) and PPAR-γ protein expression (Figure 2A), suggesting that Rh2 inhibits PPAR-γ expression at the transcriptional level and protein synthesis, and thus suppresses adipogenesis in 3T3-L1 cells.

### 2.4. Ginsenoside Rh2 Attenuates Fat Packing Proteins in 3T3-L1 Cells

**Figure 4:**
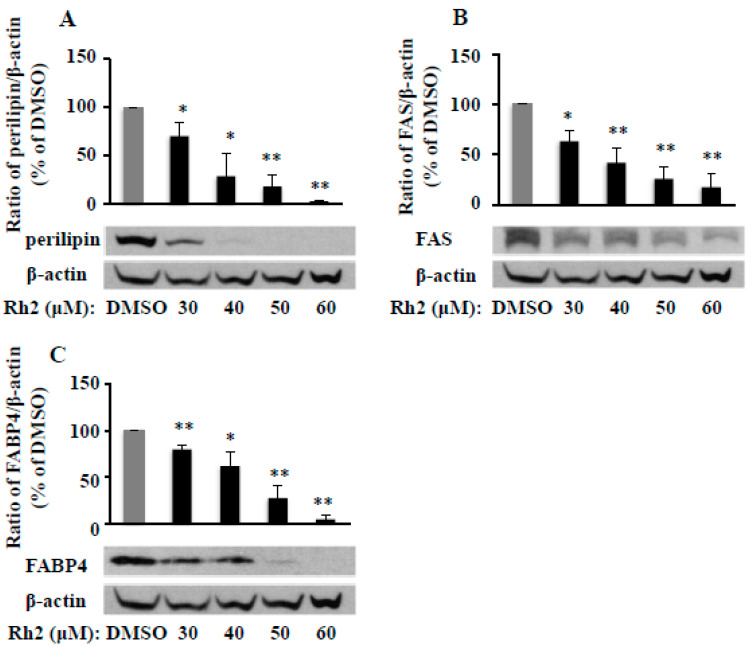
Ginsenoside Rh2 dose-dependently attenuates protein expressions of fat packing proteins perilipin (**A**), fatty acid synthase (FAS, **B**), and fatty acid binding protein 4 (FABP4, **C**) in 3T3-L1 cells. On day 7, cells treated with various concentrations of Rh2 were collected to measure perilipin, FAS, and FABP4 protein expressions by Western blot and normalized by β-actin expression. Values are means ± SE, *n* = 3. A set of representative images and bar graphs are shown. * *p* < 0.05, ** *p* < 0.01 vs. DMSO.

Fat packing is a critical step of adipogenesis, which is implemented by several packing proteins including fatty acid synthase (FAS), fatty acid binding protein 4 (FABP4), and perilipin. We found that ginsenoside Rh2 dose-dependently inhibited protein expression of perilipin (Figure 4A), FAS (Figure 4B), and FABP4 (Figure 4C) on day 7 in 3T3-L1cells. These results matched the patterns of the Rh2 inhibitory effects on fat accumulation, PPAR-γ, and C/EBP-α protein and mRNA expressions in 3T3-L1 cells, as aforementioned.

### 2.5. Ginsenoside Rh2 Dose-Dependently Inhibits Cell Differentiation in Human Primary Preadipocytes

**Figure 5:**
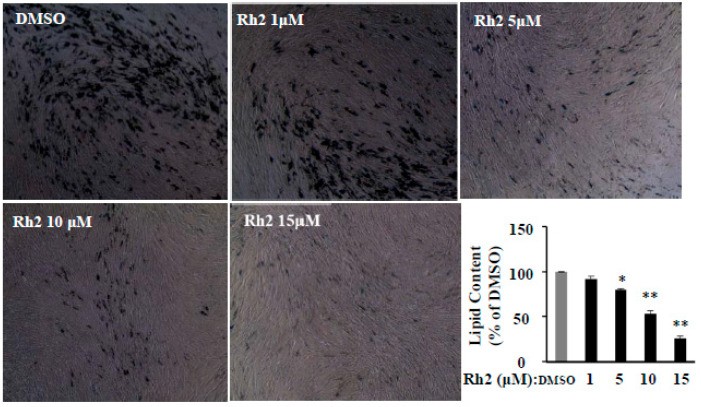
Ginsenoside Rh2 dose-dependently reverses lipid accumulation, protein expressions of differentiation markers in human primary preadipocytes (HPPs). The cells stained with Oil-red O on day 15 were dissolved in isopropanol, and fat accumulation was measured by absorbance using Synergy H1 hybrid reader at 490 nm. Oil-Red O representative images of lipid accumulation and the average bar graph are shown (40 × magnification). Values are means ± SE, *n* = 4. A set of representative images and bar graphs are shown. * *p* < 0.05, ** *p* < 0.01 vs. DMSO.

Although Rh2 dose-dependently inhibited cell differentiation and relevant key molecules’ expression in 3T3-L1 cells, the high concentrations (30–60 μM) of Rh2 and the characteristics of the 3T3-L1 cell line [21] prevent the application of Rh2 to fight obesity in humans. We recently reported that human primary preadipocytes (HPPs) were more sensitive and required much lower concentrations of the drugs than 3T3-L1 cells in the anti-adipogenic effect [22]. Indeed, compared with the effective concentrations (30–60 µM) in 3T3-L1 cells, 5–15 µM Rh2 significantly inhibited fat accumulation in HPPs (Figure 5). This was also demonstrated in the anti-adipogenic effect of ginsenoside Rg3 between HPPs and 3T3-L1 cells in our recent study [23]. These results suggest that Rh2 has the potential to exert the anti-adipogenic effect in humans.

### 2.6. Dietary Ginsenoside Rh2 Reduces Hepatic PPAR-γ Expression in Obese Mice

**Figure 6:**
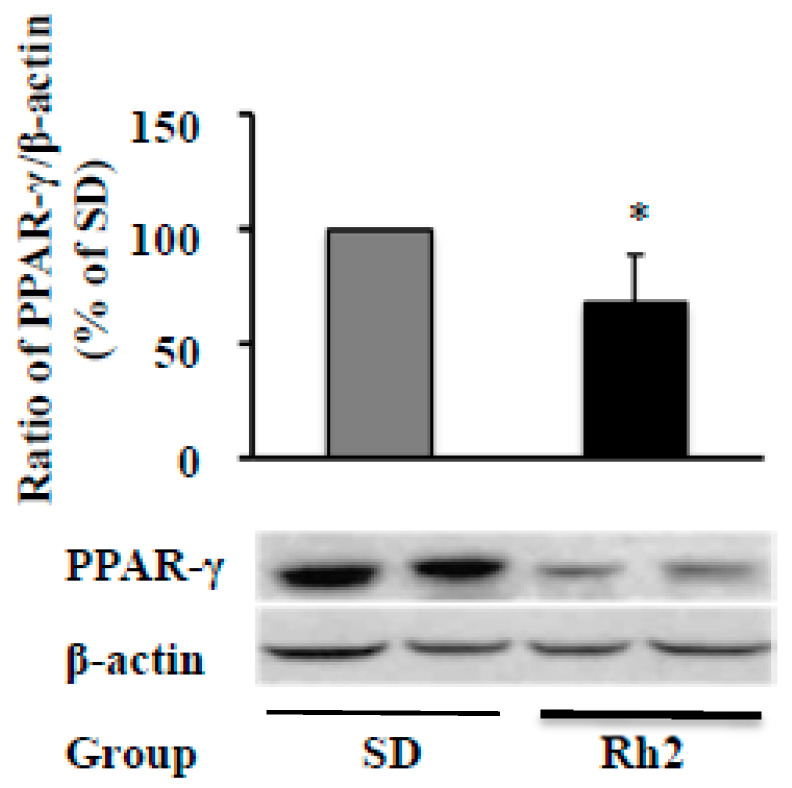
In vivo effects of dietary ginsenoside Rh2 intake. After an eight-week dietary administration of Rh2 (0.1 g/kg diet), the liver PPAR-γ protein expression in mice was determined by Western blot and normalized to β-actin expression. Values are means ± SE, *n* = 12. A set of representative images and bar graphs are shown. * *p* < 0.05 vs. standard diet (SD).

Because of the complexity of the absorption and metabolism of Rh2, it is essential to test whether dietary supplementation of Rh2 can inhibit PPAR-γ expression, and thereby prevent obesity in ob/ob obese mice, a widely used obese animal model. As shown in Figure 6A, dietary intake of Rh2 (0.1 g Rh1/kg diet, w/w, for eight weeks) significantly reduced protein PPAR-γ expression in the liver, the primary organ of adipogenesis, although we did not found a significant change of PPAR-γ expression in white adipose tissues (WATs). The fasting glucose was significantly reduced by Rh2 at the end of the experiment (Table 1), although we did not observe significant changes in the body weight, food intake, weight of the liver, and weight of WATs, as well as glucose tolerance (data not shown) by Rh2. Moreover, levels of hepatic glutathione reductase (GR) and glutathione S-transferase (GST), two major antioxidants molecules, were significantly decreased in the Rh2 group (Table 2). These enzymes are significantly increased in obese animals fed with high-fat diets and contribute to obese-induced complications. Similarly, the obese-increased hepatic GR and GST were reversed by dietary intake of grape seed extract in obese rats [24], which might be because of the oxidative stress-reducing capacity and inhibition of the generation of superoxide anions and hydroxyl-free radicals.

## 3. Discussion

In the present study, we reported for the first time, to our knowledge, that dietary supplementation Rh2 attenuated hepatic protein expression of PPAR-γ, a key regulator of adipogenesis and fat metabolism, as well as the improvements in fasting blood glucose and hepatic antioxidants GR and GST in obese mice. We also found that Rh2 dose-dependently inhibited fat accumulation and expressions of PPAR-γ, C/EBP-α, FAS, and perilipin in 3T3-L1 cells. Compared with the required high Rh2 concentrations (30–60 μM) in 3T3-L1 cells, Rh2 significantly inhibited differentiation at much lower levels (5–15 μM) in HPPs. The different effective dosages in 3T3-L1 cells and HPPs may be the result of the different characteristics of these two cells that 3T3-L1 cells are from a homogeneous population, but HPPs include both preadipocytes and fibroblast-likes cells, as we discussed [23]. These results confirm our research hypothesis that ginsenoside Rh2 inhibits adipogenesis through attenuating the PPAR-γ pathway in both preadipocytes and obese mice.

Ginsenoside Rh2 was not detected in red and black Asian ginseng, while Rg3 was increased by this process [25,26]. In American ginseng, Rh2 is not naturally present in both root and leaf; however, heat treatment (100 °C, 1.5 h) produced 11.3 ± 0.5 mg/g Rh2 in leaf, but not from heat-treated root [6]. Another study found that a total of 576 mg/g Rh2 (20(R)-Rh2 + 20(S)-Rh2) can be extracted in American ginseng leaf by the heat extracting process (100 °C, 1.5 h) [27]. However, one study found that Rh2 was not detectable in white Asian ginseng, but was detected in white American ginseng (0.007%), and long-time steaming remarkably increased Rh2 level (0.066% after 4 h steaming) in American ginseng [28]. Moreover, Rh2 can be hydrolyzed from Rg3 in the digestive system, and the maximum plasma concentration of Rh2 of 0.48 μM can be reached in 2 h after oral ginsenoside Rg3 (50 mg/kg) in normal rats [4]. In addition, this degrading process from Rg3 to Rh2 can be suppressed by a tumor in rats [4]. In humans and rats, Rh2 was transformed by intestinal bacteria from Rg3, which was metabolized in the stomach from Rb1 and Rb2, the major naturally presented ginsenosides in fresh ginseng [5,29]. Protopanaxadiol and monooxygenated protopanaxadiol are the metabolites of Rb1, Rg3, and Rh2 in rat feces [29]. Therefore, gut microbiota plays a crucial role in Rh2 conversion from Rb1 and Rg3. In addition, Rh2 can be rapidly produced from Rb1 under the acidic environment (simulated gastric fluid) [30]. Furthermore, the octyl ester derivative of Rh2 (Rh2-O) has a higher cellular uptake (63.24%) than Rh2 (36.76%) when incubated with HepG2 cells for 24 h [10]. Therefore, Rh2 is an intermediate ginsenoside from Rg3 or Rb1 by heating, intestinal bacteria, and gastric fluid, and it has a low rate of cellular uptake, but with multiple beneficial effects.

Rh2 promoted fat accumulation at low concentrations (0.01–1 μM) by activating glucocorticoid receptor in 3T3-L1 cells [17]; however, at the high level (at 20 and 40 μM), Rh2 attenuated 3T3-L1cell differentiation via activating the adenosine monophosphate (AMP)-activated protein kinase (AMPK) signaling pathway [18]. Our results are in line with this study finding that Rh2 dose-dependently (30–60 μM) inhibited fat accumulation, and accompanied by the attenuated expressions of critical markers of PPAR-γ, C/EBP-α, FAS, FABP4, and perilipin. The different effect of low level of Rh2 (1 uM) between Niu et al. [17] and our study and Hwang et al. [18] is the result of different conditions (no chemical inducer versus cocktail MDI mixture (0.5 mM IBMX, 1 μM dexamethasone, and 10 μg/mL insulin)), and the powerful MDI inducer produced strong differentiation overshadows the pro-adipogenesis effect of Rh2 at this low level. We also tested the toxicity of Rh2 using an MTT assay that all concentrations (30–60 μM) having anti-adipogenic effects are safe in 3T3-L1 cells.

AMPK is a key regulator of energy dynamics, and it produces ATP and enhances oxidative metabolism and mitochondrial biogenesis, adipogenesis, as well as lipolysis [31,32,33]. Overexpression of AMPK in mice induces the expression of genes controlling lipid oxidation and mitochondrial [34,35]. Activated AMPK may reduce PPAR-γ activity and expression in adipocytes [36], and this is supported by the study that Rh2 attenuated 3T3-L1cell differentiation via activating the AMPK signaling pathway [18]. Indeed, red ginseng [37,38,39] and Rg3, the source of Rh2, all activated AMPK activity [40,41,42]. Hence, this Rh2-activated AMPK may prevent/reduce obesity by increasing energy expenditure and inhibiting adipogenesis. Moreover, the anti-adipogenic effect of Rh2 may depend on phosphoinositide 3-kinase (PI3K)/Akt pathways and wingless-type MMTV integration site (WNT)/β-catenin pathway, which is supported by the fact that the AKT [43] and (WNT)/β-catenin pathways [44] are involved in adipogenesis and Rh2 can significantly affect these pathways [45,46].

There is no report that individual dietary Rh2 prevents obesity in animals, although red ginseng crude extract has been reported in preventing obesity in rodent models by reducing leptin level and adipogenesis level [47,48,49], as well as enhancing fatty acid oxidation and energy expenditures via activation of PPARα in rats (200 mg/kg to 10-week-old, for 32 weeks) [50]. In the present study, we found that dietary intake of Rh2 (0.01%, w/w) significantly reduced PPAR-γ protein expression in liver, fasting blood glucose, and two major endogenous antioxidant molecules GR and GST in ob/ob mice, although we did not observe significant changes of body weight, food intake, and fat pad. These results indicate that Rh2 has the potential to fight obesity, at least in the liver, the factory of fat and energy metabolism. Indeed, the prevalence rate of nonalcoholic fatty liver disease (NAFLD) increases with increasing body mass index (BMI). For instance, the prevalence rates of NAFLD are 15%, 65%, and 85% in nonobese, obese (BMI 30.0–39.9 kg/m^2^), and extremely obese patients (BMI 40 kg/m^2^), respectively [51]. However, because of the limited resources, the only one low dosage (0.01%, w/w) Rh2 was used in this animal study, which may contribute to the failure of reducing weight gain and fat pads as well as the expression of adipogenic markers including PPAR-γ, C/EBP-α, FAS, and perilipin (which have been confirmed in 3T3-L1 cells). In addition, we did not analyze the adipocyte sizes in WATs because there were no significant changes in body weight and WATs’ weight in obese mice. The Rh2 level in the blood of mice was not measured because of the limited volume of collected blood. If several different higher dosages are used in the next study, and these limitations will be solved, we can make a conclusion on whether the dietary intake of Rh2 prevents obesity in animals.

In summary, we found that Rh2 dose-dependently inhibited cell differentiation and expressions of adipogenic markers including PPAR-γ, C/EBP-α, FAS, and perilipin in 3T3-L1 cells. HPPs need much lower concentrations of Rh2 in inhibiting differentiation compared with 3T3-L1 cells. This is the first study to find that dietary supplementation Rh2 attenuated hepatic protein expression of PPAR-γ, a key regulator of adipogenesis and fat metabolism, as well as the improvements in fasting blood glucose and hepatic antioxidants GR and GST in obese mice. These results suggest that ginsenoside Rh2 dose-dependently inhibits preadipocytes differentiation through down-regulating the PPAR-γ pathway, and Rh2 may be a potential agent in preventing obesity, although more studies are needed.

## 4. Materials and Methods

### 4.1. Chemicals

The HPPs specific medium fibroblast basal medium plus fibroblast growth kit, serum-free (KS-201-040), was also obtained from ATCC (Manassas, VA, USA). Dulbecco’s modified Eagle’s medium and penicillin-streptomycin were from Gibco (Grand Island, NY, USA). Fetal bovine serum was purchased from Mediatech (Manassas, VA, USA). Ginsenoside Rh2 (99.9% purity) was purchased from ChromaDex (Irvine, CA, USA) and was dissolved in DMSO, aliquots at 100 mM, and stored in a −20 °C freezer. Insulin from Roche (Mannheim, Germany) and 3-isobutyl-1-methylxanthine (IBMX), dexamethasone, and rosiglitazone from Sigma-Aldrich (St Louis, MO, USA) were used to induce adipocyte differentiation. Specific antibodies for β-actin, C/EBP-α, FABP4, FAS, PPAR-γ, and perilipin were purchased from Cell Signaling Technology (Danvers, MA, USA). RNeasy Mini Kit was purchased from Qiagen (Valencia, CA, USA) and iTaq Universal SYBR Green One-Step Kit was purchased from Bio-Rad (Hercules, CA, USA). SuperSignal West Dura chemiluminescence kit was from ThermoFisher Scientific (Fair Lawn, NJ, USA). TACS MTT cell proliferation assay kit was purchased from Trevigen (Gaithersburg, MD, USA). Dimethyl sulfoxide (DMSO) was purchased from Sigma (St Louis, MO, USA), while 10% buffered formalin phosphate and 2-propanol were purchased from Fisher Scientific (Fair Lawn, NJ, USA). Oil-red O solution was purchased from Electron Microscopy Science (Hatfield, PA, USA).

### 4.2. Cell Differentiation

3T3-L1 preadipocytes (from ATCC, Manassas, VA, USA) were cultured in 12-well plates with Dulbecco’s modified Eagle medium (DMEM) high glucose containing 1% penicillin-streptomycin (PS) and 10% fetal bovine serum (FBS). To induce differentiation, two-day post-confluent preadipocytes were changed with cultural medium (DMEM with 1% PS and 10% FBS) supplemented with cocktail mixture (MDI: 0.5 mM IBMX, 1 μM dexamethasone, and 10 μg/mL insulin) on day 0. After three days, the cells were then maintained in normal cultural medium with 10µg/mL insulin. The 3T3-L1 cells need seven days to accomplish the differentiation process. Meanwhile, the cells were treated with various concentrations of Rh2 during the whole differentiation process from day 0 to day 7, as previously described [52]. Passages between 5 and 25 of 3T3-L1 cells were used in all experiments.

Human primary preadipocytes (HPPs, from ATCC, Manassas, VA, USA) were maintained at 37 °C and 5% CO_2_ in fibroblast basal medium plus fibroblast growth kit-serum-free (ATCC, KS-201-040) with biotin (33 μM) and pantothenate panthothenate (17 μM). After full confluent, cell differentiation (day 0) was induced with a hormone cocktail containing 0.5 mM IBMX, 1 μM dexamethasone, 1 μM rosiglitazone, and 10 μg/mL insulin in the growth medium. After three days, the medium was changed to normal growth medium containing 10 μg/mL insulin every three days until day 15. The cells were treated with various concentrations of Rh2 during the whole differentiation process from day 0 to day 15, as we described [22]. Passages between 3 and 6 of HPPs were used in all experiments.

### 4.3. Oil-Red O Staining

To determine the adipogenesis and fat accumulation in the adipocytes, 3T3-L1 cells (day 7) and HPPs (day 15) were stained with Oil-red O. The cells were gently washed with phosphate-buffer saline (PBS) once and fixed with 10% formalin for 1 h at 4 °C. The fixed cells were washed with PBS three times. The cells were then stained with filtered Oil-red O solution for 30 min at room temperature and then washed five times with water to remove the unstained Oil-red O solution. Fat droplets in the adipocytes were stained as red; images were taken under an optical microscope. The relative fat accumulation has dissolved the dye in 100% isopropanol and measured using a Synergy H1 hybrid reader (BioTek Instruments, Inc. Winooski, VT) at 490 nm, as we described [22].

### 4.4. Cell Viability Assay

To test whether Rh2 directly kills cells at the selected concentrations, TACS MTT cell proliferation assay (Trevigen, Gaithersburg, MD, USA) was conducted to evaluate cell viability according to the manufactory’s instruction, as we described [23]. Briefly, 3T3-L1 cells were seeded in 12-well plates (about 20% confluent) with different dosages of Rh2 from 30 μM to 300 μM for 72 h. Cells were then incubated with MTT reagent (100 μL per well) for 4 h and added detergent reagent (500 μL each well) and incubated in the dark for 4 h. The relative cell viability was determined by the absorbance at 570 nm using a Synergy H1 hybrid reader (BioTek Instruments, Inc. Winooski, VT).

### 4.5. Animals

Four-week-old male obese leptin-deficient mice (C57BL/6J-Lep^ob^, ob/ob, Jackson Laboratory, Bar Harbor, ME, USA) were randomly divided into two groups (12 mice/group), which were fed with either AIN-93G mineral mix standard food (powder) [53], or standard food containing 0.01% ginsenoside Rh2 (0.1 g Rh2/kg diet, w/w, mixed Rh2 with standard powder food, and fed in food jar) for eight weeks. This dosage was calculated based on previous whole ginseng extracts in animals [47,48,49,50], and our in vitro studies (0.5% whole ginseng extracts × 20% of Rb1 on the whole extracts = 0.01%). The detailed compositions of diets are listed in Table 2. All animals were maintained at constant temperature and humidity with a 12:12 h light/dark cycle permitted consumption of water and food ad libitum. Bodyweight and food intake were measured weekly throughout the study. At the end of the study, all mice were fasted overnight and euthanized with CO_2_, according to the American Veterinary Medical Association guidelines. Blood, liver, and fat tissues were collected and prepared for blood glucose, antioxidant activity, and relevant biomarkers analyses. All experimental procedures were approved by the Institutional Animal Care and Use Committee at Tennessee State University following the National Institutes of Health Guidelines for the Care and Use of Laboratory Animals.

### 4.6. Protein Extraction

At the end of the differentiation process with/out treatments, 3T3-L1 cells and HPPs were collected to extract proteins, as we described [23]. Briefly, cells were rinsed once with PBS and added with mammalian protein extraction buffer. Cells were then scraped off and collected in 1.5 mL tube and sonicated on ice for 15 s three times, with a 15 s interval. After centrifuging 10 min at 12,000× *g*, the supernatant was collected as a protein sample. Sample protein concentration was measured using a Pierce BCA protein assay kit. An equal amount of protein of samples was mixed with 2× Western blot sample buffer and heated at 95 °C for 5 min, and then subjected to Western blot analysis.

Animal tissues liver or white adipose tissues (WATs) collected from the in vivo study were weighted (about 50 mg each mouse) and added in mammalian protein extraction buffer with proteinase inhibitor cocktail (Sigma-Aldrich). After cutting 30 times, samples were homogenized by the Precellys 24 tissue homogenizer on ice three times, 15 s each time. Then, samples were centrifuged 10 min at 12,000× *g*, and supernatants were collected as protein sample. For fat tissue, about 20 mg fat was put into the specific adipose tissue protein extraction buffer (HEPES, 50 mM; NaCl, 150 mM; glycerol, 10%; Triton X-100, 1%) with proteinase inhibitor cocktail (Sigma-Aldrich). After 30 times cutting, samples were homogenized by Precellys 24 tissue homogenizer on ice three times, 15 s each time. Then, samples were centrifuged 20 min at 18,000× *g* at 4 °C. After removing the fat cake from the top, the supernatant was transferred to a new tube as a protein sample. Sample protein concentration was measured using a Pierce BCA protein assay kit. The samples were directly used to measure antioxidant levels as below. For Western blot, the normalized protein samples were mixed with 2 × Western blot sample buffer and heated at 95 °C for 5 min, and then subjected to Western blot analysis.

### 4.7. Western Blot

Protein samples from cultured cells and animal tissues were analyzed by Western blot, as we described [23]. Briefly, samples were separated by 10% SDS-PAGE, and the membrane was blocked for 1 h with Tris-buffered saline-Tween (TBST) containing 5% skim milk at room temperature. After washing 3 times with TBST, the membrane was incubated with the relevant primary antibody at 4 °C overnight. On the next day, the membrane was washed three times with TBST and then incubated with secondary antibody for one hour at room temperature. Specific bands were detected by SuperSignal West Dura chemiluminescence (ThermoFisher Scientific), and visualization was performed by exposure of the membranes to X-ray films. Band intensities were quantified by ImageJ software. The value of the specific protein was normalized by the expression of β-actin and expressed as the percentage of the control.

### 4.8. Total RNA Isolation and Quantitative Real-Time PCR Analysis

At the end of the adipogenic process, total RNA from 3T3-L1 adipocytes was isolated using the RNeasy Mini Kit (Qiagen, Valencia, CA, USA), as we described [23]. According to the manufacturer’s instructions, 30 ng sample of total RNA was applied in the real-time quantitative PCR (SYBR green) analysis using a Bio-Rad iTaq Universal SYBR Green One-step Kit. Designed primers were as follows: PPAR-γ forward: TCG CTG ATG CAC TGC CTA TG, PPAR-γ reverse: GAG AGG TCC ACA GAG CTG ATT [54]; β-actin forward: AGC CTT CCT TCT TGG GTA TGG, β-actin reverse: CAC TTG CGG TGC ACG ATG GAG [55]. The optimized reaction cycles: cDNA synthesis 10 min at 50 °C, reverse transcriptase inactivation 5 min at 95 °C, 45 cycles for PCR and detection, 10 s at 95 °C, and 30 s at 60 °C. Relative gene expression was normalized by the β-actin expression and calculated using the 2^−ΔΔCt^ method.

### 4.9. Hepatic Antioxidants

Extracted liver protein samples from above were used to evaluate levels of antioxidants glutathione reductase (GR) and glutathione S-transferase (GST) using a colorimetric detection assay kit according to the manufacturer’s instructions (Cayman Chemicals). Briefly, the samples were treated with metaphosphoric acid and triethanolamine to remove protein to avoid interferences owing to particulates and sulfhydryl groups on proteins in the assay. Then, 50 µL of the sample was added with assay cocktail and incubated for 25 min; concentrations of GR and GST were measured at 405 nm and calculated from the standard curve, as previously described [56]. Samples were tested in duplicate using Synergy H1 hybrid reader (BioTek Instruments, Inc. Winooski, VT). The value was normalized by the protein level and expressed as nmol/min/ mg protein.

### 4.10. Statistical Analyses

All values of in vitro studies were presented as means ± SEM of at least three independent experiments performed in triplicate. For the animal study, 12 mice/group was determined by conducting a power analysis (using GPOWER software) for a one-way analysis of variance (ANOVA) at a type error level of 5%. The values of in vivo studies were presented as means ± SEM of 12 mice. All data were analyzed with one-way ANOVA and significant differences between treatment groups were further analyzed using Tukey test. Differences with a *p*-value < 0.05 were considered significant.

## Figures and Tables

**Table 1:** Bodyweight gain, food intake, weight of liver, weight of white adipose tissues (WATs), fasting blood glucose, and hepatic antioxidants in obese mice.

Group	Body Weight Gain (g/mouse)	Food Intake (g/mouse/day)	Liver Weight (g)	WATs Weight (g)	Fasting Blood Glucose (mg/dL)	Liver Glutathione Reductase (nmol/min/mg protein)	Liver Glutathione S-Transferase (nmol/min/mg protein)
SD	21.6 ± 1.85	3.85 ± 0.30	1.78 ± 0.22	2.71 ± 0.31	160 ± 16.9	50.2 ± 3.77	1171 ± 53.3
Rh2	24.1 ± 0.82	3.82 ± 0.30	1.67 ± 0.23	2.69 ± 0.39	132 ± 11.4 *	38.7 ± 0.91 *	1044 ± 17.2 *

Values are means ± SE, *n* = 12. * *p* < 0.05 vs. standard diet (SD).

**Table 2:** Compositions of standard AIN-93G diet and Rh2 supplemented diet.

Ingredient	Standard Diet (g/kg diet)	Rh2 Supplemented Diet (g/kg diet)
Cornstarch	466	466
Casein (>85%protein)	140	140
Dextrinized cornstarch (>90% tetrasaccharides)	155	155
Sucrose	100	100
Soybean oil	40.0	40.0
Fiber	50.0	50.0
Mineral mix (AIN-93M-MX)	35.0	35.0
Vitamin mix (AIN-93-vx)	10.0	10.0
L-Cystine	1.80	1.80
Choline bitartrate	2.50	2.50
Ter-butyhydroquinone	0.008	0.008
Ginsenoside Rh2	0.00	0.10
Total	1000	1000

## Abbreviations

BMI	body mass index
C/EBP-α	CCAAT/enhancer binding protein-alpha
DMEM	Dulbecco’s modified Eagle’s medium
DMSO	dimethyl sulfoxide
FAS	fatty acid synthase
FABP4	fatty acid binding protein 4
GR	glutathione reductase
GST	glutathione S-transferase
HPPs	human primary preadipocytes
IBMX	3-isobutyl-1-methylxanthine
MDI medium	IBMX + dexamethasone + insulin medium
PBS	phosphate-buffered saline
PCR	polymerase chain reaction
PPAR-γ	peroxisome proliferator-activated receptor-gamma
WATs	white adipose tissues
